# The Impact of Postmastectomy Radiation Therapy on the Outcomes of Prepectoral Implant-Based Breast Reconstruction: A Systematic Review and Meta-Analysis

**DOI:** 10.1007/s00266-022-03026-y

**Published:** 2022-07-25

**Authors:** Abdelrahman Awadeen, Mohamed Fareed, Ali Mohamed Elameen

**Affiliations:** 1 Department of Plastic and Reconstructive Surgery, Faculty of Medicine (Boys), Al-Azhar University, Al Mokhaym Al Daem, Gameat Al Azhar, Nasr City, Cairo, Egypt; 2 Department of Plastic and Reconstructive Surgery, El-Sahel Teaching Hospital, Cairo, Egypt

**Keywords:** Radiotherapy, Radiation, Prepectoral, Implant, Breast reconstruction

## Abstract

**Background:**

Breast reconstruction is the mainstay treatment choice for patients subjected to a mastectomy. Prepectoral implant-based breast reconstruction (IBBR) is deemed to be a promising alternative to subpectoral reconstruction. Postmastectomy radiation therapy (PMRT) is necessary for locoregional recurrence control and to improve the disease-free survival rate in locally advanced breast cancer. This systematic review and meta-analysis study was designed to reveal the surgical, aesthetic, and oncological outcomes of prepectoral IBBR after PMRT.

**Methods:**

An extensive literature search was performed from inception to March 28, 2022. All clinical studies that included patients who were subjected to prepectoral IBBR and PMRT were included. Studies that included patients who received radiation therapy before prepectoral IBBR were excluded.

**Results:**

This systematic review included six articles encompassing 1234 reconstructed breasts. Of them, 391 breasts were subjected to PMRT, while 843 breasts were not subjected. Irradiated breasts were more susceptible to develop wound infection (RR 2.49; 95% 1.43, 4.35; *P* = 0.001) and capsular contracture (RR 5.17; 95% 1.93, 13.80; *P* = 0.001) than the non-irradiated breasts. Furthermore, irradiated breasts were more vulnerable to losing implants (RR 2.89; 95% 1.30, 6.39; *P* = 0.009) than the non-irradiated breast. There was no significant difference between both groups regarding the risk of implant extrusion (RR 1.88; 95% 0.20, 17.63; *P* = 0.58).

**Conclusions:**

Patients with prepectorally IBBR and PMRT were more vulnerable to developing poor outcomes. This included a higher risk of breast-related and implant-related adverse events.

**Level of Evidence III:**

This journal requires that authors assign a level of evidence to each article. For a full description of these Evidence-Based Medicine ratings, please refer to the Table of Contents or the online Instructions to Authors www.springer.com/00266.

**Supplementary Information:**

The online version contains supplementary material available at 10.1007/s00266-022-03026-y.

## Background

Breast reconstruction is the mainstay treatment choice for patients subjected to a mastectomy. It aimed to restore the breast mound and maintain the patients’ well-being without negatively affecting breast cancer prognosis. Implant-based breast reconstruction (IBBR) is the most performed restorative technique following mastectomy. In the USA, approximately 80% of patients seeking breast reconstruction are subjected to IBBR, in contrast to 18% to autologous reconstruction [1]. IBBR is associated with favorable aesthetic outcomes, a low complication rate, and reasonable affordability. Throughout the past era, IBBR techniques have evolved dramatically from complete submuscular coverage to partial muscular coverage. However, subpectoral implant placement is associated with muscle spasm, animation deformity, severe postoperative pain, and surgical morbidity. The desire for women to recreate a natural breast with less pain and minimal downtime increased the need for less invasive IBBR [2, 3].

Prepectoral IBBR is deemed to be a promising alternative to subpectoral reconstruction. Adopting the acellular dermal matrix (ADM) has offered implant-support soft tissue coverage. This product has made prepectoral breast reconstruction safe and reproducible [4]. Prepectoral IBBR involves filling the gap between the mastectomy skin flap and pectoralis major muscle. This technique eliminates the need for elevation and dissection of the pectoralis muscle, adjacent muscles, and facia. This preserves the pectoralis major muscle in its anatomical position, resulting in a more natural breast appearance and less postoperative pain [5–7]. Additionally, prepectoral reconstruction minimizes the risk of animation deformity, implant lateralization, and discomfort resulting from muscle spasms [8].

Radiation therapy is required for nearly 40% of patients subjected to mastectomy. Postmastectomy radiation therapy (PMRT) is necessary for locoregional recurrence control and to improve the disease-free survival rate in locally advanced breast cancer [9, 10]. Despite these therapeutic advantages, PMRT is associated with devastating consequences in the IBBR. PMRT decreased the quantity and quality of microvascular blood supply to the breast. This ischemia decreases the integrity of the skin flaps and increases the fibrosis and scarring of breast tissue [11, 12]. Soft tissue changes induced by PMRT are challenging to be corrected, resulting in permanent unacceptable cosmetic outcomes [13, 14]. Despite these devastating complications, PMRT remained a necessary treatment for patients subjected to breast reconstruction [15].

Despite the advantages of prepectoral IBBR, challenges remained with this procedure in the PMRT setting. Most published studies assessed the utility of PMRT after subpectoral reconstruction, and few have existed for prepectoral reconstruction. The published evidence related to these outcomes is inconclusive and contradictory [16, 17]. The desire of surgeons and oncologists to achieve acceptable cosmetic results while maintaining oncological safety highlighted the need to reveal the impact of PMRT on the outcomes of prepectoral IBBR. Therefore, this systematic review was designed to summarize the data reported in the literature on the surgical, aesthetic, and oncological outcomes of prepectoral IBBR after PMRT. Such evidence is mandated to alleviate the repercussions of PMRT by adopting timely and effective care for patients subjected to prepectoral IBBR.

## Methods

This systematic review was carried out following the Preferred Reporting Items for Systematic Reviews and Meta-Analyses (PRISMA) guidelines [18] and the Cochrane collaboration recommendations [19] (Supplementary Table.1). The study's methodology was documented in a protocol registered in the PROSPERO database (number; CRD42022311635).

### Data Source

An extensive literature search was performed from inception to March 28, 2022, using the following databases: PubMed, Google Scholar, Web of Science (ISI), SIGLE, Scopus, Virtual Health Library (VHL), Clinical trials, NYAM, Controlled Trials (mRCT), EMBASE, Cochrane Collaboration, and WHO International Clinical Trials Registry Platform (ICTRP). No restrictions were employed on patients’ age, sex, ethnicity, language, race, or place.

The search strategy implemented controlled vocabulary terms under the criteria of each searched database. The medical subject headings and text words were used to ensure that a considerable range of relevant articles were evaluated. The following keywords were used in every possible combination: 'Radiotherapy,' 'Radiation,' 'Prepectoral,' 'Breast,' 'Mammary,' 'Reconstruction.' A further manual search was performed to distinguish all additional conceivable articles that were not indexed.

### Study Selection

All clinical studies that included patients who were subjected to prepectoral IBBR and PMRT were included. No restrictions were implemented on the patient’s age, sex, race, or place. Studies that included patients who received radiation therapy before prepectoral IBBR were excluded. Furthermore, studies in which data were unattainable to be extracted, review articles, non-human studies, guidelines, case reports, letters, editorials, posters, comments, and book chapters were excluded. Two reviewers performed the title, abstract, and full-text screening process to disclose the potentially relevant articles that met the eligibility criteria. The discussion dissolved the contradiction between the reviewers. The screening process and the causes of article exclusion were documented using PRISMA flowchart.

### Data Extraction

Two reviewers extracted the data in a well-structured Microsoft excel spreadsheet. The following study characteristics data were extracted from the finally included articles; the title of the included studies, the second name of the first author, publication year, study design, study period, and study region. Baseline patients' demographic characteristics were extracted, including the sample size, number of breasts, age, ethnicity, race, body mass index (BMI), and comorbidities. The data related to breast cancer and surgical procedures were extracted. The breast-related adverse events and implant-related side effects were extracted. The functional and oncological outcomes were evaluated.

### Quality Assessment

The quality of the retrospective studies was estimated using the National Institute of Health (NIH) quality assessment tool [20]. The studies were assorted into good, fair, and bad when the score was <65%, 30-65%, and> 30%, respectively.

### Statistical Analysis

The risk ratio (RR) and confidence interval (95% CI) were used for analyzing dichotomous variables. The fixed-effect model was implemented when a fixed population effect size was assumed. Otherwise, the random-effects model was used. Statistical heterogeneity was estimated using Higgins *I*^2^ statistic, at the value of > 50%, and the Cochrane Q (*Chi*^2^ test), at the value of *p* < 0.10 [21]. Data analysis was performed using Review Manager version 5.4 [22]. The significant difference was established at the value of *P* < 0.05.

## Results

**Fig. 1:**
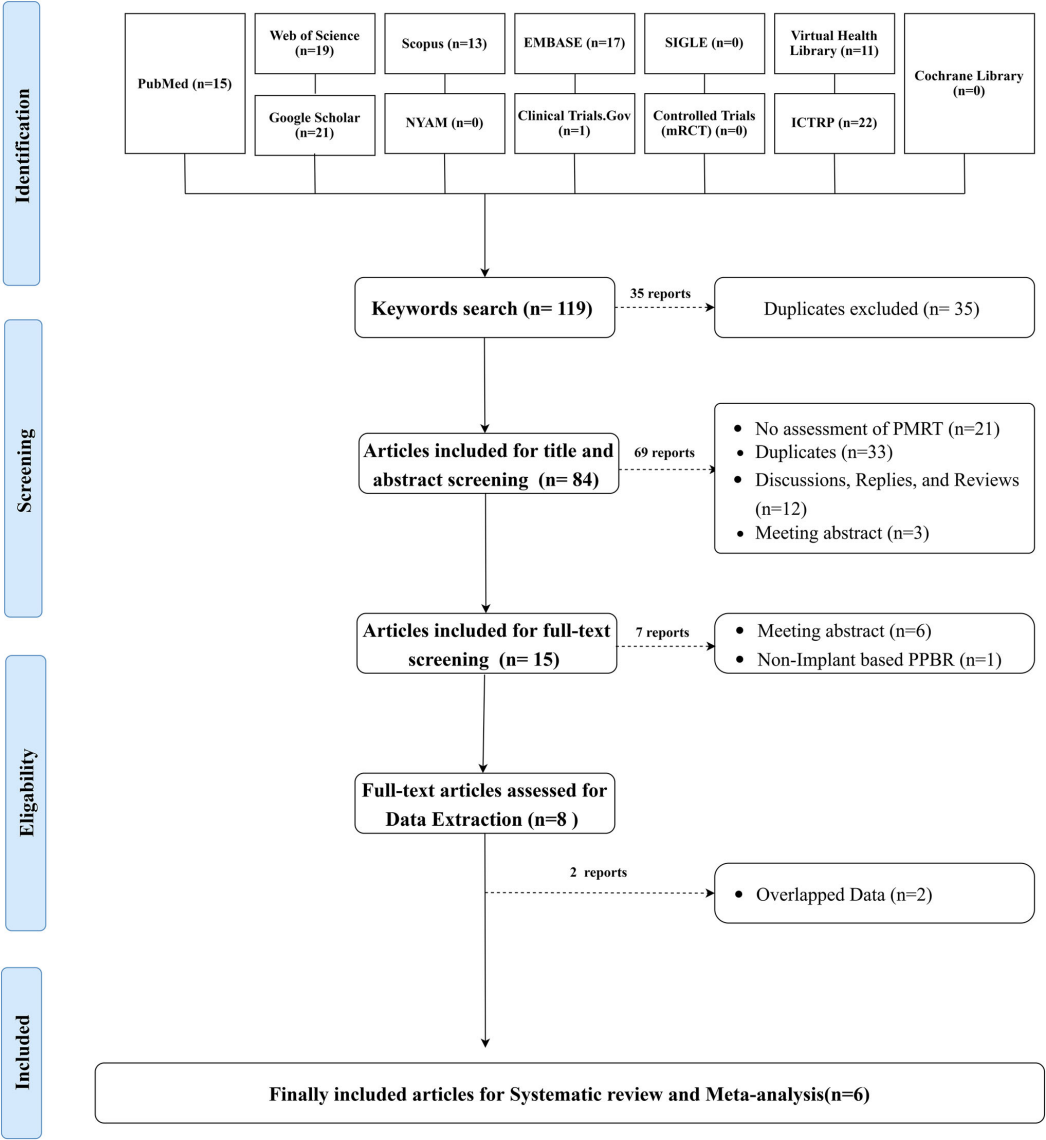
PRISMA flowchart showing the process of the literature search, title, abstract, and full-text screening, systematic review, and meta-analysis

The literature review yielded 119 articles. Out of them, 35 reports were duplicates, revealing 84 articles eligible for screening. Screening of the title and abstract revealed 15 articles eligible for full-text screening. Of them, eight articles were included for data extraction. Two articles were excluded being overlapped data, revealing six articles eligible for systematic review. The keywords used for each searched database are shown in Supplementary Table 2. The processes of searching strategy, screening, and eligibility are shown in the PRISMA flowchart (Fig. 1).

### Baseline Demographic Characteristics and Quality Assessment

This systematic review included six articles encompassing 1234 reconstructed breasts [23–28]. Of them, 391 breasts were subjected to PMRT, while 843 breasts were not subjected. All the included studies were retrospective designs. The average age ranged from 46.6 to 55.6 years among patients in the irradiated group and from 50.6 to 53.4 years among the non-irradiated group. Out of the included patients, 65 patients were current smokers, while 49 patients had diabetes mellitus. The average follow-up period ranged from 6 to 60.7 months. Based on the NIH quality assessment tool, the included studies were of good quality (Table.1).

**Table 1:** Baseline demographic characteristics of the included studies

Study ID		Study region	Study design	Study period	Sample size		Number of breasts		Age (years)	
					Irradiated	Non-irradiated	Irradiated	Non-irradiated	Irradiated	Non-irradiated
					Number	Number	Number	Number	Mean ±SD	Mean ±SD
1	Elswick et al. [23]	USA	Retrospective study	October 2012 to December of 2016	54		39	93	48 (30–69)*	
2	Polotto et al. [24]	Italy	Retrospective study	January 2015 to September 2018	28	158	28	174	55.6±10.8	53.4±10.4
3	Sbitany et al. [25]	USA	Retrospective study	2015 to 2017	NR	NR	175		46.6 ± 10.2	
4	Sigalove et al. [26]	USA	Retrospective study	August 2014 to May 2016	33		34	18	50.6 ± 12.1	
5	Sinnott et al. [27]	USA	Retrospective study	January 1, 2010, and December 31, 2019	45	305	71	493	53.5 ± 11.3	52.3 ± 9.5
6	Thuman et al. [28]	USA	Retrospective study	June 2012 to August 2019	24	34	44	65	NR	NR

Study ID		BMI (kg/m^2^)		Current smokers		Hypertension	Diabetes mellitus	Follow-up period	Quality assessment	
		Irradiated	Non-irradiated	Irradiated	Non-irradiated					
		Mean ±SD	Mean ±SD	Number	Number	Number	Number		%	Decision
1	Elswick et al. [23]	27.2 (19.4-40.7)		0		9	0	19 (1–36)*	83.33	Good
2	Polotto et al. [24]	23.5±3.3	23.6±3.85	3	20	NR	NR	6.1–60.7	83.33	Good
3	Sbitany et al. [25]	24.5 ± 5.1		4		NR	6	9.0 ± 6.1	75	Good
4	Sigalove et al. [26]	27.7 ± 5.9		12		2	13	25.1±6.4	66.66	Good
5	Sinnott et al. [27]	29.8 ± 6.2	28.5 ± 5.9	4	21	NR	19	22.3 ± 17.6	75	Good
6	Thuman et al. [28]	30.3	27.73	0	1	NR	11	6	75	Good

*NR* non-reported

^*^Data reported in the form of median and range

Three studies included patients with breast cancer stage < IV. There were 218 and 289 patients with unilateral and bilateral breast cancer, respectively. The average radiation dosage ranged from 46 to 60 Gy with an average duration of 35–246 days. Furthermore, 178 patients were subjected to nipple-sparing mastectomy. Two-stage prepectoral IBBR was performed among 416 reconstructed breasts, while 321 received adjuvant lipofilling (Table2).

**Table 2:** dfasfsf

Study ID		Breast cancer stage	Side of breast cancer				Chemotherapy			Radiation dose
			Unilateral		Bilateral		Neoadjuvant only	Neoadjuvant and adjuvant	Adjuvant only	
			Irradiated	Non-irradiated	Irradiated	Non-irradiated				
			Number	Number	Number	Number	Number	Number	Number	
1	Elswick et al. [23]	II, III, and IV	39		15		31	2	13	50 Gy in 25 fractions (range, 49–60 Gy in 25–30 fractions).
2	Polotto et al. [24]	<IV	NR	NR	NR	NR	0	29	61	46–50 Gy in 2.0 Gy per fraction
3	Sbitany et al. [25]	II and III	NR	NR	NR	NR	57	0	23	5000 cGy given in 180–200 cGy
4	Sigalove et al. [26]	NR	14		19		NR	NR	NR	NR
5	Sinnott et al. [27]	NR	26	108	19	197	46			50 Gy in 2-Gy daily fractions
6	Thuman et al. [28]	NR	12	19	16	23	NR	NR	NR	NR

Study ID		Radiation duration	Oncological procedures						Breast reconstruction approach	Adjuvant lipofilling	
			Nipple-sparing mastectomy		Skin-sparing mastectomy		Areola-sparing mastectomy				
			Irradiated	Non-irradiated	Irradiated	Non-irradiated	Irradiated	Non-irradiated		Irradiated	Non-irradiated
			Number	Number	Number	Number	Number	Number		Number	Number
1	Elswick et al. [23]	NR	18	16	35	22	1	1	Two-stage	42	31
2	Polotto et al. [24]	142.29 days (range, 60–246 days)	141		NR	NR	NR	NR	Immediate	NR	NR
3	Sbitany et al. [25]	NR	3		49		0	0	Two-stage	NR	NR
4	Sigalove et al. [26]	NR	NR	NR	NR	NR	NR	NR	Immediate, direct-to-implant or two-staged	NR	NR
5	Sinnott et al. [27]	5 days per week for 5–6 weeks	NR	NR	NR	NR	NR	NR	Immediate, direct to implant, two-stage	31	207
6	Thuman et al. [28]	NR	NR	NR	NR	NR	NR	NR	Two-stage	NR	NR

*Gy* Gray, *NR* Non-reported

### Breast-Related Adverse Events

#### Wound Infection and Dehiscence

**Fig. 2:**
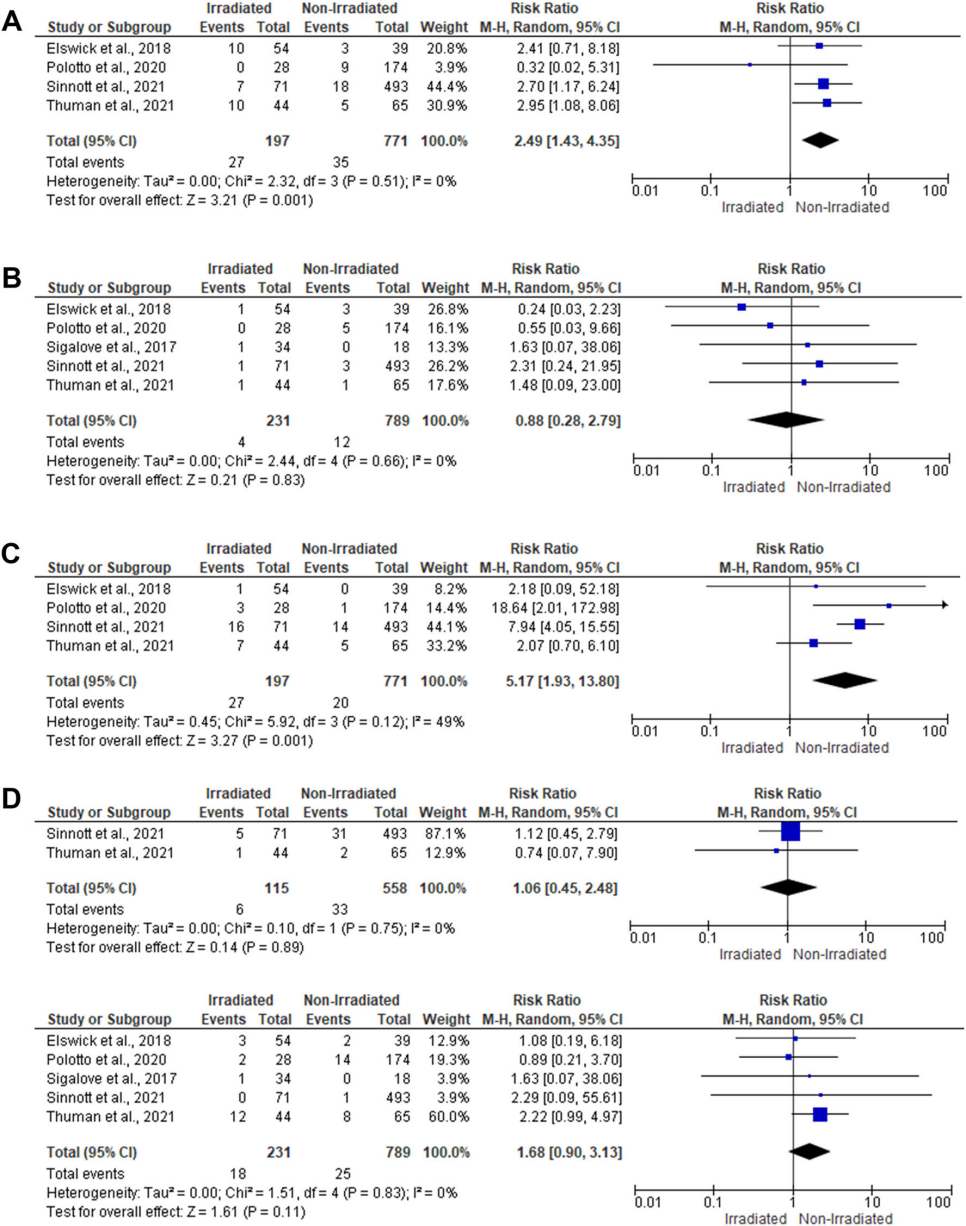
Forest plot of summary analysis of the risk ratio and 95% CI of **a** the risk of wound infection between the irradiated and the non-irradiated breasts. **b** The risk of wound dehiscence between the irradiated and the non-irradiated breasts. **c** The risk of capsular contracture between the irradiated and the non-irradiated breasts. **d** The risk of nipple necrosis between the irradiated and the non-irradiated breasts. **e** The risk seroma between the irradiated and the non-irradiated breasts. Size of the blue squares is proportional to the statistical weight of each trial. The black diamond represents the pooled point estimates. The positioning of both diamonds and squares (along with 95% CIs) beyond the vertical line (unit value) suggests a significant outcome (IV = inverse variance)

The risk of wound infection was evaluated within four articles [23, 24, 27, 28], including 968 reconstructed breasts. In the random-effects model (*I*^2^ = 0%, *P* = 0.51), irradiated breasts were 2.49 times more susceptible to develop wound infection (RR 2.49; 95% 1.43, 4.35; *P* = 0.001), relative to the non-irradiated breasts. Five studies reported the wound dehiscence risk within 1020 reconstructed breasts [23, 24, 26, 27]. There was no significant difference between the irradiated and the non-irradiated breasts (RR 0.88; 95% 0.28, 2.79; *P* = 0.83) (Fig. 2a, b).

#### Capsular Contracture and Nipple Necrosis

Four studies [23, 24, 27, 28], including 968 reconstructed breasts, evaluated the capsular contracture risk between the irradiated and the non-irradiated breasts. In the random-effects model (*I*^2^ = 49%, *P* = 0.12), irradiated breasts were 5.17 times more vulnerable to developing capsular contracture than the non-irradiated breasts (RR 5.17; 95% 1.93, 13.80; *P* = 0.001). The nipple necrosis risk was assessed within two studies [27, 28] , including 673 reconstructed breasts. There was no risk difference between the irradiated and the non-irradiated breasts (RR 1.06; 95% 0.45, 2.48; *P* = 0.89) (Fig. 2c, d).

#### Seroma and Hematoma

**Fig. 3:**
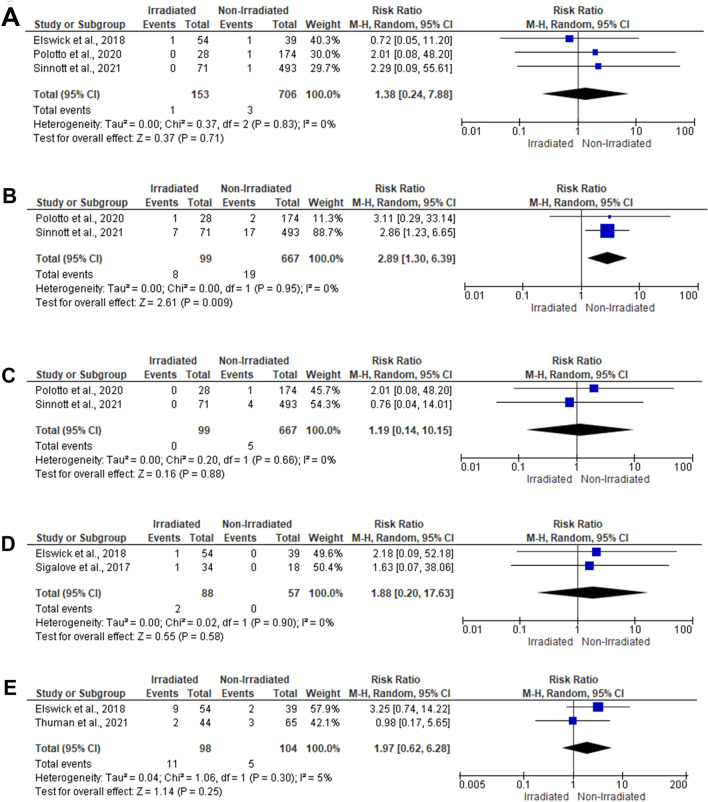
Forest plot of summary analysis of the risk ratio and 95% CI of **a** the risk of hematoma between the irradiated and the non-irradiated breasts. **b** The risk of implant loss between the irradiated and the non-irradiated breasts. **c** The risk of breast rippling between the irradiated and the non-irradiated breasts. **d** The risk of implant extrusion between the irradiated and the non-irradiated breasts. **e** The risk of device explanation between the irradiated and the non-irradiated breasts. Size of the blue squares is proportional to the statistical weight of each trial. The black diamond represents the pooled point estimates. The positioning of both diamonds and squares (along with 95% CIs) beyond the vertical line (unit value) suggests a significant outcome (IV = inverse variance)

The risk of seroma was evaluated among 1020 reconstructed breasts within five studies [23, 24, 26, 27]. In the random-effects model (*I*^2^ = 49%, *P* = 0.12), there was no significant difference between the irradiated and the non-irradiated breasts (RR 1.68; 95% 0.90, 3.13; *P* = 0.11). There was no significant risk difference between the irradiated and the non-irradiated breasts regarding the risk of hematoma (RR 1.38; 95% 0.24, 7.88; *P* = 0.71) (Figs. 2e and 3a).

### Implant-Related Adverse Events

The impact of PMRT on the risk of implant loss was evaluated within two studies [24, 27], including 766 reconstructed breasts. Pooling the data revealed that irradiated breasts were 2.89 times more vulnerable to losing implants (RR 2.89; 95% 1.30, 6.39; *P* = 0.009) compared to non-irradiated breasts. The risk of breast rippling was reported in two studies [24, 27], including 766 reconstructed breasts. There was no significant risk difference between the irradiated and the non-irradiated breasts (RR 1.19; 95% 0.14, 10.15; *P* = 0.88). There was no significant difference between the irradiated and the non-irradiated breasts regarding the risk of implant extrusion (RR 1.88; 95% 0.20, 17.63; *P* = 0.58). Two studies included 202 reconstructed breasts reported the risk of device explanation in prepectoral IBBR after PMRT. In the random-effects model (I^2^ = 5%, *P* = 0.30), there was no significant difference between the irradiated and the non-irradiated breasts (RR 1.97; 95% 0.62, 6.28; *P* = 0.25) (Fig. 3b–e).

## Discussion

Prepectoral IBBR in the PMRT setting presents a unique challenge. This is because of the devastating consequences of PMRT on the soft tissue envelopes around the implant in the absence of vascularized muscle coverage [29]. Whereas many published reports revealed the promising results of prepectoral IBBR, the outcomes in the PMRT setting deserved further evaluation. This is because of the lack of well-structured randomized clinical trials and prospective studies that revealed these outcomes [30–32]. Therefore, this systematic review and meta-analysis was executed to ascertain the aesthetic, functional, and oncological outcomes of prepectoral IBBR in the PMRT.

This study revealed poor aesthetic and surgical outcomes among patients with prepectoral IBBR and PMRT. This included a significantly higher rate of wound infection, capsular contracture, and implant loss. There was no difference between both groups regarding the risk of seroma, hematoma, implant extrusion, and device explanation. The findings of the present systematic review were concomitant with previous studies. El‐Sabawi et al. reported a high rate of total complications, reoperation, and reconstruction failure in prosthetic reconstruction after radiation [33]. In this respect, Lam et al. reported poor cosmetic outcomes and high reconstruction failure rates in immediate breast reconstruction after adjuvant radiotherapy [34].

Radiation represents the most deliberating factor for IBBR. PMRT causes acute toxicity in the form of inflammation, edema, and desquamation. These changes lead to wound infection, dehiscence, seroma, and delayed healing [35]. Radiation therapy induces microvascular occlusion, altering the vascularity of the overlying skin flap for placement of prepectoral expanders. Expansion against inadequately vascularized skin flap increases the risk of flap necrosis, implant exposure, and extrusion [36, 37]. Irradiated breasts release transforming growth factors, leading to chronic tissue changes. This includes atrophy and fibrosis of the skin and underlying subcutaneous tissues, resulting in skin discoloration, retraction, induration, and decreased breast volume. Furthermore, PMRT can induce soft tissue necrosis, resulting in capsular contracture, implant loss, and distortion of the breast contour after reconstruction [34, 38]. In consistent with these findings, Zugasti et al. [39] reported a higher rate of early and late complications among patients subjected to PMRT after immediate IBBR. They reported a lower satisfaction rate and poor cosmetic outcomes associated with PMRT.

Noteworthy, ADM provides a safe barrier supporting the prosthesis in the IBBR. ADM diminishes the profibrotic and inflammatory responses, increasing the biointegration of implants and decreasing the capsular contracture risk [40]. In the present study, the risk of capsular contracture was approximately fivefold among the irradiated breasts in comparison with the non-irradiated. This finding highlighted that the ADM might be less beneficial in the PMRT. In particular, the skin reaction to PMRT is not eliminated by the protective function of ADM, leading to thickening and fibrosis of the skin envelope [41]. This finding was parallel with Valdatta et al. [42], who reported a negative impact of radiation therapy on breast reconstruction even with ADM use.

Fat grafting may have an integral role in improving the status of the skin envelope and shaping the skin flap. Early fat grafting improves tissue perfusion and healing by the capitalization of tissues for graft regeneration and retention [43]. In the setting of prepectoral IBBR, adjuvant lipofilling was performed to improve the thickness of the mastectomy flap and to recontour breast defects after PMRT [23, 27]. The timing of radiotherapy may influence the outcomes of prepectoral IBBR. The delivery of PMRT after complete recovery and healing from the surgical interventions can minimize the risk of skin necrosis and wound dehiscence [44, 45]. Paradoxically, Momoh et al. reported a comparable oncological and surgical outcomes pre and after radiation therapy in the IBBR [16]. The volume of the implant may attribute to the complications associated with PMRT after prepectoral IBBR [24]. Given the fact that radiotherapy is a main line in treating patients with breast cancer, prospective investigations are needed to detect the methods needed to prevent the devastating impact of radiation on the prepectoral IBBR. Polotto et al. [24] reported a relatively high dissatisfaction rate with breasts among patients with irradiated breasts using the BREAST-QTM. This dissatisfaction was reflected in the physical, psychological, and sexual well-being of patients with irradiated breasts. Sinnott et al. [27] reported a relatively higher locoregional recurrence rate among patients with irradiated breasts. There was a similar rate of distant metastasis among patients with irradiated and non-irradiated breasts. Many factors contribute to the complications following PMRT after IBBR. This includes the patient’s demographic, tumor characteristics, reconstructive indications, the timing of reconstruction, implant characteristics, and adjuvant therapies. Therefore, further studies are necessary to predict the long-term functional and oncological outcomes of prepectoral IBBR in the setting of PMRT [46, 47].

The current systematic review consolidated the evidence related to the impact of PMRT on the prepectoral IBBR. Conversely, some limitations should be considered. The included studies were retrospective designs, revealing a risk of information selection bias. There was heterogeneity between the included studies. Such heterogeneity may be evolved because of the difference in patients' characteristics, reconstruction methods, assessment methods, radiation protocols, and follow-up intervals.

## Conclusions

Patients with prepectorally IBBR and PMRT were more vulnerable to developing poor outcomes. This included a higher risk of breast-related and implant-related adverse events. Recognizing these devastating complications should raise the awareness of plastic surgeons and oncologists to optimize the possible preventive measures to minimize the complications and maintain oncological outcomes in patients undergoing IBBR and receiving PMRT.

## Supplementary Information

Below is the link to the electronic supplementary material.

Supplementary file1 (DOCX 29 kb)
